# The impact of cochlear implantation on cognition in older adults: a systematic review of clinical evidence

**DOI:** 10.1186/s12877-015-0014-3

**Published:** 2015-02-25

**Authors:** Gina Miller, Craig Miller, Nicole Marrone, Carol Howe, Mindy Fain, Abraham Jacob

**Affiliations:** The University of Arizona Speech, Language, and Hearing Sciences, Tucson, AZ 85724 USA; Department of Otolaryngology - Head & Neck Surgery, The University of Arizona College of Medicine, Tucson, AZ 85724 USA; Arizona Health Sciences Library, University of Arizona College of Medicine, Tucson, AZ 85724 USA; The University of Arizona College of Medicine, Arizona Center on Aging, Tucson, AZ 85724 USA; Department of Otolaryngology - Head & Neck Surgery, The University of Arizona Ear Institute, The University of Arizona College of Medicine, Tucson, AZ 85724 USA; The University of Arizona Cancer Center, The University of Arizona Bio5 Institute, 1515 N. Campbell Ave., P.O. Box 245024, Tucson, AZ 85724 USA

**Keywords:** Cognition, Cognitive decline, Cognitive impairment, Elderly, Elderly people, Hearing impairment, Hearing loss, Older people, Cochlear Implant

## Abstract

**Background:**

Hearing loss is the third most prevalent chronic condition faced by older adults and has been linked to difficulties in speech perception, activities of daily living, and social interaction. Recent studies have suggested a correlation between severity of hearing loss and an individual’s cognitive function; however, a causative link has yet to be established. One intervention option for management of the most severe to profound hearing loss in older adults is cochlear implantation. We performed a review to determine the status of the literature on the potential influence of cochlear implantation on cognition in the older adult population.

**Methods:**

Over 3800 articles related to cochlear implants, cognition, and older adults were reviewed. Inclusion criteria were as follows: (1) study population including adults > 65 years, (2) intervention with cochlear implantation, and (3) cognition as the primary outcome measure of implantation.

**Results:**

Out of 3,886 studies selected, 3 met inclusion criteria for the review.

**Conclusions:**

While many publications have shown that cochlear implants improve speech perception, social functioning, and overall quality of life, we found no studies in the English literature that have prospectively evaluated changes in cognitive function after implantation with modern cochlear implants in older adults. The state of the current literature reveals a need for further clinical research on the impact of cochlear implantation on cognition in older adults.

## Background

The purpose of this systematic review is to provide an overview of existing literature on the association between hearing loss and cognition among older adults and summarize the state of available clinical evidence on the impact of cochlear implantation on cognition. Hearing loss is one of the most common human sensory disabilities. Older adults are dramatically affected by hearing loss, with prevalence increasing with advancing age. Several population-based studies of US adults over age 65 years estimate hearing loss prevalence at 42-47% in one or both ears [1,2], and nearly 90% of those over age 80 years have at least a mild hearing loss. [2]. Presbycusis, or age-related hearing loss, is typically sensorineural, progressive, affects both ears, and is sometimes associated with central auditory deficits and tinnitus [3,4]. Hearing loss has become the third most common chronic health condition faced by older adults [1], and its prevalence is expected to increase as the aging population continues to grow rapidly [5]. The impact of acquired hearing loss in older adults is far-reaching, including communication difficulties, social isolation, depression, an association with falls and declines in physical functioning, and decreased quality of life [6,7].

### Hearing loss and cognition

As cognition is the mental process of acquiring knowledge and understanding through thought, experience, and the senses, hearing impairment would be expected to impact cognition [8]. For decades it has been recognized that auditory acuity affects performance on verbal and non-verbal cognitive assessments, with a number of theoretical accounts put forward to explain the relationship age-related changes have in hearing and cognition [9,10]. Yet only recently has a link been established between hearing impairment and risk of cognitive decline.

Several recent publications by Lin and colleagues [11,12] support the notion that the severity of hearing loss in older adults is independently linked to accelerated cognitive decline. In a prospective study examining the Baltimore Longitudinal Study of Aging cohort, an independent association was found between hearing loss and dementia in adults over 60 years of age [11]. Subsequently, Lin and colleagues investigated this link among adults between the ages of 70 to 79 years enrolled in the Health ABC (Health, Aging, Body Composition) study. Results confirmed the significant correlation between a greater level of hearing loss and poorer cognitive function on both verbal and non-verbal cognitive tests [12]. During the 6-year testing period, adults with hearing loss exhibited a 30% to 40% accelerated rate of cognitive decline and a 24% increased risk for incident cognitive impairment compared to normal hearing older adults. Those with severe hearing loss were at five times the risk of developing dementia as those with normal hearing. If the link between hearing loss and cognitive decline is both causal and directional, improving hearing through audiologic rehabilitation strategies such as hearing aids or cochlear implants could mitigate the cognitive decline associated with sensory degradation and trajectories associated with advancing age.

### Management of acquired severe to profound hearing loss

There are a variety of management options available to people with hearing loss, including counseling and education about alternate communication techniques, use of hearing aids and assistive listening devices, as well as use of surgically placed amplification devices such as middle ear implants and cochlear implants. Hearing aids have been shown to somewhat improve cognitive abilities and to reduce listening effort [13,14]. Unfortunately, with more severe to profound degrees of hearing loss, conventional hearing aids often increase auditory awareness without substantially improving speech discrimination or communicative ability [5].

Among many older individuals with greater degrees of hearing loss and poor speech recognition abilities, cochlear implantation might offer a more effective intervention as compared to hearing aids [15,16]. Cochlear implants are small, implanted electronic devices that consist of a sound processor that sits behind the ear, a transmitter that is held on the skull with a magnetic coil, a receiver/stimulator implanted under the skin of the skull, and an electrode array that is coiled within the cochlea. The microphone delivers sound to the speech processor, which processes the acoustic signal into an electrical signal and delivers it to the transmitter. The transmitter sends the signal, via the magnet on the skull, to the receiver/stimulator, which sends the electrical pulses to the electrode array in the cochlea. These electrodes collect the impulses and deliver them to the auditory nerve.

Candidacy for cochlear implantation is determined by a combination of audiologic and medical evaluations. Age is not a contraindication. Cochlear implantation is typically a ninety-minute procedure performed in ambulatory settings under general anesthesia and now routinely done for those persons of advanced age [17]. Potential candidates must demonstrate limited benefit from properly fitted hearing aids on standardized speech perception tests in a sound booth without visual cues [18]. Most private insurance companies allow implantation in patients with < 50% speech understanding ability in the ear to be implanted with < 60% for the contralateral ear and binaural condition. Medicare has more stringent criteria for coverage, requiring speech-understanding to be < 40% in the ear to be implanted and in the binaural condition. Compared with individuals fit with hearing aids, cochlear implant recipients show twice the improvement in overall quality of life measures, in part because pre-operative ratings of quality of life are lower due to the greater degree of hearing loss before the intervention [19].

### Cochlear implants and older adults

Only 5-10% of adult cochlear implant candidates in the US have received cochlear implants, despite the fact that Medicare and many insurance carriers currently pay for the procedure [20,21]. Approximately 50,000 cochlear implant surgeries are currently performed per year worldwide based on manufacturer revenue estimates, and about 20–25,000 are used in adults. The most rapidly growing segment receiving cochlear implants are those over age 65 (http://www.medel.com/cochlear-implants-facts/ and personal communication with FDA approved CI manufacturers, Abraham Jacob, MD). The average delay between onset of severe to profound hearing loss and the receipt of a cochlear implant in adults is approximately 10 years [22]. Should there be a direct and causal link between hearing loss and cognitive decline, a population experiencing the most severe degree of auditory deprivation over time would be an important group in which to study the question.

To assess the current status of the literature on the cognitive outcomes of cochlear implantation in older adults, we reviewed the medical and psychological literature for studies investigating cognitive abilities in post-lingually deafened older adult cochlear implant recipients. In this systematic review, we will present a summary of existing empirical evidence and then discuss current theoretical accounts of the links between audition and cognition.

## Methods

A literature review was planned and performed using methods specified in the Preferred Reporting Items for Systematic Reviews and Meta-Analyses (PRISMA) guidelines [23]. Initial search terms were compiled and iteratively refined by content experts in the fields of Otology, Neurotology, & Cranial Base Surgery; Speech, Language, and Hearing Sciences; Library Science and Geriatrics. Both controlled vocabulary terms (e.g. MeSH) and key words were used to search the following databases for articles related to cochlear implants, cognition, and older adults: PubMed/MEDLINE (1946–2014), Wiley/Cochrane Library (1898–2014), Thomson-Reuters/Web of Science (1898–2014) EBSCO/PsycINFO (1880’s-present), and EBSCO/CINAHL(1981–2014). Literature searches were completed in March, 2013. The complete PubMed/MEDLINE Search strategy, upon which the other database searches were also built, is available in Appendix A. Reference lists of citations to the ultimately included articles were also searched for articles that might meet inclusion criteria.

Inclusion criteria were (1) Population: the study had to include at least some individuals aged ≥ 65 years. (2) Intervention with cochlear implantion (i.e. studies that looked only at hearing aids were excluded). (3) Outcomes including assessment of cognition or cognitive processing (i.e. studies that looked only at Quality of Life parameters or spoken language outcomes were excluded).

Two independent reviewers performed the study selection (CM, GM). In case of disagreements, a third reviewer (AJ) cast the deciding vote. Titles and abstracts of retrieved references were screened for inclusion and full texts of potential articles were further analyzed to see if they met inclusion criteria. Case reports, letters, and systematic reviews were excluded. After inclusion, study characteristics, research goals and findings with respect to cochlear implantation and cognition in older adults were reviewed and analyzed.

## Results

**Figure 1:**
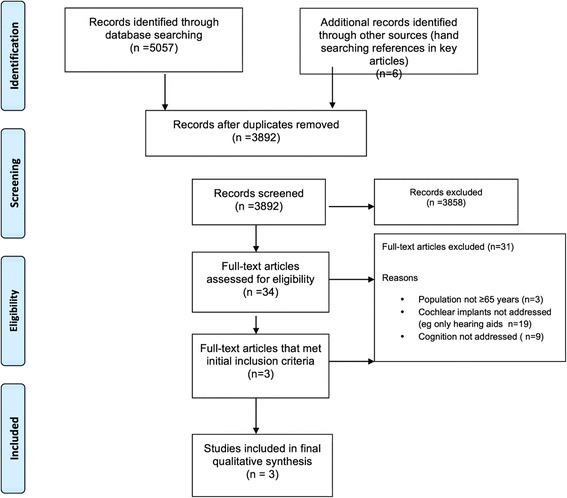
**Flowchart of the process of literature search and extraction of studies meeting the inclusion criteria.**

We found 5057 articles through database searching and 6 additional articles through citation analysis of the most relevant articles. Of the 3892 articles which remained after duplicates were removed, 3858 were excluded because of irrelevance to the topic (Figure 1). Strict inclusion/exclusion criteria as outlined above were applied to 34 articles. Of these, only 3 studies [24-26] met the full criteria for population including adults ≥ 65 years, using cochlear implants, and outcomes evaluating cognition rather than solely quality of life, psychosocial parameters, or hearing and spoken language outcomes (Figure 1).

The three studies that met criteria for the review included cognitive measures in older adults, but were inconclusive in terms of cognitive benefit provided by cochlear implantation. Table 1 summarizes the three studies. Although these studies show evidence that quality of life for individuals with cochlear implants is significantly improved when compared to those with hearing aids, effects on cognition (either positive or negative) were not shown.

**Table 1:** **Summary of studies that examined the impact of cochlear implantation on cognition in older adults**

**Study**	***N***	**Age range**	**Cochlear implant**	**Pre-implant cognitive measures**	**Cognitive outcome measures**	**Outcome**
Vega (1977) [24]	13	23-67 years	Single-electrode	None	No tests depended on auditory functioning. Duration of implant use not reported. Used sub-tests of Halsted-Reitan neuropsychological test battery (spatial & symbolic abilities, visual temporal acuity, speed of response, attention and test motivation, and intellectual and conceptual functioning), Continuous reaction time test, Purdue pegboard, Trails test, Weschler-Bellvue Form II test	Average scores were within range of general population. 11 CI recipients with normal to superior cognitive functioining; 2 CI recipients with impaired cognitive and conceptual functioning
Crary, Wexler, Berliner, & Miller (1982) [25]	46	19-75 years	Single-electrode	WAIS; MFD; Trlmk; BNDR; BIP	Same as pre-implant measures. Tested one-year post-CI (n = 23) and at two years or more post-CI (n = 16).	As a group, scores in low range of normal intelligence and results stable over time. Process of CI candidacy screened out individuals with cognitive/psychological disorder.
Aplin (1993) [26]		14-80 years	Nucleus 22-channel	WAIS-R or WISC-R	Details not reported.	No adverse effects reported. Described following subjects at intervals post-implant to monitor for improvements in functioning; however, methods and results not reported.

*Note.* CI = Cochlear implant; WAIS = Wechsler Adult Intelligence Scale; MFD = Graham-Kendall Memory for Designs; Trlmk = Trail Making Test; BNDR = Bender Visual Motor Gestalt; BIP = Bender Visual Motor Interference; WAIS-R = Wechsler Adult Intelligence Scale- Revised; WISC-R = Wechsler Intelligence Scale for Children.

The 1977 study by Vega [24] assessed neuropsychological status of 13 subjects with a mean age of 48 years (range 23 to 67) having single-channel cochlear implants. Tests used were subtests of the the Halstead-Reitan neuropsychological test battery, screening tests for brain damage, and parts of the Wechsler-Bellevue Form II test. Several of the tests used in this study compare right and left-sided functioning, which is thought to be sensitive to specific neurological problems. Pre-implantation studies were not performed; therefore, changes in cognition pre- versus post-implantation could not be discerned. Post-implant test results found that 11 scored within normal limits and 2 demonstrated impaired cognitive and conceptual abilities.

Crary et al. [25] analyzed psychometric data obtained from 46 postlingually deafened adults who underwent cochlear implantation with a single-channel device in order to assess psychological and cognitive effects of cochlear implantation. The ages of the subjects ranged from 19 to 75 years with a mean age of 48 years. The cognitive tests used were: Wechsler Adult Intelligence Scale (WAIS) assessing IQ and intellectual function, Graham-Kendall Memory for Designs (MFD) to measure immediate recall, Trail Making Test (Trlmk) assessing the ability to maintain a cognitive set and while engaging in a task, as well as Bender Visual Motor Gestalt (BNDR), and Bender Visual Motor Interference Test (BIP) to evaluate visual motor functions. Cognitive testing was performed pre-implantation for all subjects; follow-up testing was done one year post-implantation; and, for some subjects at two years or more post-implantation. The results indicated that there was no damage in cognitive function post-implantation. The evidence also showed that the subjects continued to pursue their daily activities as efficiently as they had pre-implantation. Numerous individuals showed improvement in several of the cognitive tests, which was thought to be a direct effect of cochlear implantation.

Aplin [26] examined the psychological status pre- and post-implantation of 30 adult recipients of a multi-channel cochlear implant with profound post-lingual hearing loss. The age range of subjects was 14 to 80 years with a mean age of 49 years. Several measures were used in this study to assess reading level, intellectual ability, personality, listening ability, and self-reported benefit due to implantation. Reading level was assessed using the British Ability Scales Word Reading Test. The main cognitive assessment tool used was The Wechsler Adult Intelligence Scale-Revised (WAIS-R) or the Wechsler Adults Intelligence Scale for Children-Revised for those under the age of 16 years. Results from the study revealed that subjects reported improvements in their communication abilities early after implantation. There were no adverse effects on intellectual ability, personality, or motivation. It should be noted that the participants in this study, pre-implantation, exhibited a wide range of scores on intellectual and personality measures, all within normal limits. The small number of subjects and lack of long-term follow-up in this study did not allow for a definitive conclusion about group performance post-implantation in regards to cognition.

## Discussion

From this systematic review, we conclude that there is both need and rationale for well-designed studies that assess cognitive outcomes and monitor whether elderly cochlear implant recipients modify their expected trajectories for cognitive decline based on rehabilitating severe to profound hearing loss. Despite an extensive search of the literature, our review identified only three dated studies that considered neurocognitive outcomes following cochlear implantation in adults over age 65 years. Our finding was surprising, given the remarkable importance of cognitive health in successful aging and known interactions between auditory perception and cognitive processes including focused attention, executive functions, learning, and memory [27]. Knutson and colleagues observed that the limited research in this area might be attributed in part to controversy as to how to assess psychological variables in deaf individuals and in part due to challenges in test administration of standardized assessments with this population [28].

Within the three studies reviewed here, the primary rationale for the examination of cognitive outcomes was to document any adverse effects of cochlear implant surgery itself on global intellectual ability. Surgical procedures in cochlear implantation have far advanced since these early studies, and it is now well established that cochlear implantation is a safe surgical procedure across the lifespan [29-31]. For example, stimulator-receivers are now less bulky, surgical tools for cochlear insertions are refined, operative magnification/lighting have improved, and electrode arrays are thinner; all coming together to allow for smaller incisions and dramatically shortened operative times as compared to earlier generations of technology. Conclusions from these studies must also be limited because the research has not specifically focused on cognitive outcomes or the older adult population in adequate sample sizes with control groups. The cognitive abilities and measures included in studies to date have focused on measures of intelligence. In future work, researchers may consider additional measures that relate to fluid cognitive abilities, such as working memory, processing speed, and attention.

Conclusions from the three extant studies are also limited by the fact that the participants received technology that would now be considered obsolete. Individuals in the Vega [24] and Crary et al. [25] studies were implanted with single-channel devices, which would have provided a far poorer representation of auditory signals as compared to modern multi-channel arrays. Individuals in the study by Aplin [26] used a multi-channel electrode; yet the patterns of neural activation for speech with this implant would be relatively more coarse in spectral and temporal representation as compared to today’s cochlear implant technology. For example, Dowell reviewed the evidence supporting the effectiveness of cochlear implants in adults across the past several decades and found that average open-set sentence performance averaged less than 40% for sound processors in the 1990s as compared to on average 80% correct scores with modern technology, even without visual cues. Signal processing in cochlear implant sound processors, approaches to electrical stimulation and electrode design, as well as surgical techniques continue to improve with each generation of technology [32].

Although the impact of cochlear implantation on cognitive processes in older adults has been relatively unexplored, its efficacy as a medical treatment and its impact on spoken language understanding has been well documented with this age group. Clark and colleagues [5] discussed the literature evaluating cochlear implant rehabilitation in patients over 65 years of age. They found that implantation was associated with improved communication, social participation, and quality of life outcomes. However, their work did not review literature specifically examining the effects of cochlear implantation on cognitive function in older adults.

Cognitive function has also been evaluated as a predictor variable in studies of factors that affect open-set speech recognition with cochlear implants with children [27,33,34] and adults [35-37]. Holden and colleagues (2013) examined the outcomes of 114 postlingually deaf adults age 23–83 years who received cochlear implants between 2003–2008. A cognitive test battery was given to all individuals *pre-operatively*, including measures of short-term and working memory, language, and reasoning/executive function. The authors suggested that cochlear implantation might stimulate cognitive function. However, postoperative cognitive testing was not performed, and to our knowledge, direct tests of this hypothesis have yet to be to be reported [37].

The neurophysiological mechanisms of cognitive decline and hearing loss remain largely unknown; however, numerous hypotheses have been put forth over time. For example, the information degradation hypothesis presented by Pichora-Fuller suggests that a combination of changes in both hearing and cognitive function affect the ability of older adults to listen, comprehend, and communicate in difficult listening environments [38]. In her studies, cognitive performance was tested using measures of memory, comprehension, attention, and speed of processing under different listening conditions. Overall, the results indicated that increased listening effort had adverse effects on the cognitive measures.

The auditory scaffolding hypothesis described by Conway and colleagues posits that sound may provide scaffolding, or a supportive framework, for cognitive abilities related to processing time and serial order behaviors [39]. Under conditions of auditory deprivation, neural reorganization is hypothesized to result in disruption of cognitive sequencing abilities. This hypothesis was formulated based on their findings that normal hearing adults perform best on sequencing tasks when the sense of hearing rather than vision is used. Data from large pediatric cochlear implant programs indicate that auditory deprivation may have modality-specific effects on central processing operations for auditory input only as well as modality-general effects on central processing for various stimuli including auditory, visual, and even tactile inputs [40]. Such effects may also occur in patients with prolonged auditory deprivation as older adults. Lastly, people are social creatures with a need for human interaction. Stated eloquently by Helen Keller, “blindness separates people from things; deafness separates people from people.” The isolation and interpersonal withdrawal resulting from hearing loss may itself impact higher executive functioning [17].

Other potential explanations for the empirically observed co-variation in hearing and cognitive function [11,12] include (1) the common cause hypothesis [10,41] and (2) accumulated impairment hypothesis. The former suggests that deterioration in both sensory and cognitive domains may reflect common insults that functionally impair the aged brain while the latter argues that changes to sensory inputs accumulate over time to structurally or functionally alter brain functions. Lindenberger and Baltes demonstrated that sensory function, especially vision and hearing, predicted age related variance in cognitive abilities [10]. While noting that their findings could not distinguish between the possible underlying mechanisms, a common cause for age related changes in brain function across cognition, audition, and vision does not exclude the possibility that perceptual changes impact cognitive processing directly. For example, Anstey and colleagues hypothesized that age related sensory changes may result in impaired sensory coding that causally impairs performance on cognitive tasks [42]. Anstey postulated that while a common factor explains most of the shared variance among cognition, age, processing speed, and sensory function, significant unique effects were not shared. In fact, an independent effect of hearing on cognition was observed. If sensory acuity were simply a proxy for aging [43], empirical studies demonstrating correlations between sensory acuity and cognitive function might not be causally linked. However, the concept that accumulated sensory insults lead to impaired brain function is line with modern concepts of neural plasticity.

The impact of significantly improving one sensory modality – hearing – and its impact on cognitive function may be uniquely tested by longitudinally assessing cognition in cochlear implant recipients. Such studies have not been published to date. We argue that this represents an important gap in scientific and clinical knowledge and a bottleneck to future progress in mediating the association between hearing and cognitive decline in older adults.

## Conclusions

Cognitive decline among older adults is multifactorial, and frequently devastating to patients and families. In normal cognitive aging, most adults over 65 years will not develop dementia or mild cognitive impairment [44]. Thus, older adults with untreated moderate to profound hearing loss may needlessly suffer from potentially preventable cognitive impairment, and cochlear implant rehabilitation may provide a reasonable alternative. However, based on our review of literature revealing only 3 articles published on the topic, we conclude that there is a lack of meaningful published data on the effects of aural rehabilitation with cochlear implants on cognitive function in older adults. Further studies are needed to better understand the relationship between hearing and cognitive function, and provide guidance for optimizing the management of severe hearing loss in older adults with cochlear implants.

## Appendix A

The following search strategy was used in the PubMed database: "Cognitive Hearing Science" OR "Auditory Cognitive Science" OR "Cochlear Implants"[Mesh] OR "Cochlear Implantation"[Mesh] OR "Hearing Loss/surgery"[Mesh] OR "Deafness/surgery"[Mesh] OR "Correction of Hearing Impairment"[Mesh] OR (cochlear implant*) OR (cochlear prosthe*) OR (auditory prosthe*) AND "Health Services for the Aged"[Mesh] OR "Aged"[Mesh] OR "Aging"[Mesh] OR "Age Factors"[Mesh] OR "Geriatrics"[Mesh] OR "Medicare"[Mesh] OR elder[tw] OR elders[tw] OR elderly[tw] OR old[tw] OR older[tw] OR aged[tw] OR aging[tw] OR ageing[tw] OR senior[tw] OR seniors[tw] OR medicare[tw]AND cognit* OR learn* OR memor* OR attent* OR "information process*" OR "language process*" OR "executive function*" OR dement* OR "mental process*" OR think* OR thought* OR understand* OR "Cognition"[Mesh] OR "Cognition Disorders"[Mesh] OR "Mental Processes"[Mesh] OR "Dementia"[Mesh].

Search strategies applied in the other databases (Cochrane Library, Web of Science, PsycINFO, and CINAHL) were derived from the PubMed search. The database search was conducted without using language or publication date restrictions.
